# Association Between the Neutrophil-To-Lymphocyte Ratio and Diabetes Secondary to Exocrine Pancreatic Disorders

**DOI:** 10.3389/fendo.2022.957129

**Published:** 2022-07-22

**Authors:** Guanhua Chen, Chunlu Tan, Xubao Liu, Yonghua Chen

**Affiliations:** Department of Pancreatic Surgery, West China Hospital of Sichuan University, Chengdu, China

**Keywords:** diabetes mellitus, neutrophil-to-lymphocyte ratio, exocrine pancreatic disorders, inflammation index, pancreatic adenocarcinoma

## Abstract

**Background:**

Diabetes mellitus among patients with exocrine pancreatic disorders is commonly known to be associated with chronic inflammation, including chronic pancreatitis and pancreatic ductal adenocarcinoma (PDAC). The neutrophil-to-lymphocyte ratio (NLR) is a novel marker that indicates the presence of various chronic inflammatory diseases, including type 2 diabetes (T2DM). However, no studies have examined the relationship between the NLR value and diabetes secondary to exocrine pancreatic disorders.

**Aim:**

To determine whether the NLR value is associated with diabetes secondary to exocrine pancreatic disorders.

**Methods:**

The medical data of subjects with confirmed pancreatic disease who were admitted to the Department of Pancreatic Surgery of our institution from August 2017 to October 2021 were obtained from the database and retrospectively analyzed. Anthropometric measures, laboratory data, including HbA1c, fasting insulin, and fasting C-peptide levels and the inflammatory index (white blood cell count, NLR, platelet-to-lymphocyte ration, monocyte-to-lymphocyte ratio) were recorded. The NLR is the ratio of neutrophils to lymphocytes. A homeostasis model (HOMA-B and HOMA-IR) was used to measure beta-cell dysfunction and insulin resistance.

**Results:**

The NLR values of the diabetes secondary to exocrine pancreatic disorders group were significantly higher than those of the nondiabetic group (P=0.001). In multivariate logistic regression, after adjusting for covariates, high NLR values were found to be an independent risk factor for diabetes secondary to exocrine pancreatic disorders (OR: 1.37, 95% CI: 1.138-1.649, P=0.001). According to Spearman correlation analysis, the NLR was significantly correlated with fasting plasma glucose levels (P<0.0001) and HOMA2-IR values (P=0.02).

**Conclusion:**

The NLR inflammation marker was significantly higher in subjects with diabetes secondary to exocrine pancreatic disorders and was associated with insulin resistance. NLR values may be reliable predictive markers for diabetes among patients with exocrine pancreatic disorders.

## Introduction

Type 3c diabetes mellitus (T3cDM), termed pancreatic or pancreatogenic diabetes and resulting from underlying exocrine pancreatic disease, accounts for 5-10% of all diabetic patients in Western populations (1). Unlike type 1 diabetes mellitus(T1DM), which is characterized by autoimmune inflammation, or type 2 diabetes mellitus (T2DM), which is characterized by insufficiency of insulin secretion and insulin resistance, T3cDM affects all subtypes of islet β cells, leading to endocrine and exocrine dysfunction of the pancreas. T3cDM is heterogeneous and involves multifactorial underlying mechanisms consisting of the inflammation, fibrosis, and sclerosis of pancreatic tissue (2).

Prior studies have identified an association between an altered immune system and T2DM, and chronic inflammation has been implicated as a potential contributor to the development of DM and its complications (3–5). The neutrophil-to-lymphocyte ratio (NLR), a repeatable and affordable indicator of inflammation derived from peripheral blood, has been discussed as a potential biomarker to reflect the inflammatory status. Previous researchers have identified its role in the occurrence and progression of T2DM and T2DM-related complications (4, 6). Moreover, many studies found that the NLR was also an effective indicator for the severity or prognosis of pancreatic diseases, including acute pancreatitis and pancreatic cancer (7, 8). However, no studies have examined the relationship between the NLR and pancreatic diabetes. Therefore, we aimed to explore the association between the NLR and DM secondary to exocrine pancreatic disorders.

## Methods

The medical database of patients with pancreatic disease who were admitted to the Department of Pancreatic Surgery, West China Hospital, during the period from August 2017 to October 2021 was retrospectively analyzed after approval of the institutional board. The inclusion criteria were as follows: patients with pancreatic disease confirmed by postoperative pathology and patients with pancreatic disease with plasma HbA1c levels and fasting plasma glucose (FPG) levels measured before surgery. Patients with acute inflammatory diseases, infectious diseases, immune disorders, glucagonomas, insulinomas, hepatic failure, previously diagnosed DM, serum creatinine levels >120 µmol/L, or those receiving hormone therapy were excluded. According to the criteria for inclusion, 291 patients were analyzed in this study, 100 of whom were diagnosed with pancreatic benign and low-grade tumors (PBLT), 45 of whom were diagnosed with chronic pancreatitis (CP), and 146 of whom were diagnosed with pancreatic ductal adenocarcinoma (PDAC).

Information on the participants’ demographics (age, sex, ethnicity), height and weight was collected from computerized records and databases. Body mass index (BMI) was calculated by dividing weight in kg by height in m^2^. Laboratory tests were performed within several minutes after blood samples were obtained before surgery. Plasma HbA1c, FPG, serum urea, creatinine, triglyceride, albumin, HDL and LDL cholesterol levels were obtained and recorded from the patient file system. We also recorded the hemoglobin level, white blood cell count, lymphocyte count, platelet count, neutrophil count, and monocyte count. An NLR, monocyte-to-lymphocyte ratio (MLR) or platelet-to-lymphocyte ration (PLR) value was obtained by dividing the number of neutrophils, monocytes, or platelets by the number of lymphocytes. A homeostasis model (HOMA-B and HOMA-IR) was used to measure beta-cell dysfunction and insulin resistance. DM is defined as a fasting blood glucose level (FBG) >7.0 mmol/L and a glycated hemoglobin (HbA1c) level > 6.5% or a postprandial blood glucose level (PBG) >11.1 mmol/L according to the 2010 American Diabetes Association guidelines.

### Statistics Analysis

All statistical analyses were carried out using Statistical Package for Social Sciences (SPSS) Version 26. Categorical variables were expressed as percentages (%) and continuous variables were expressed as the mean ± SD or median and interquartile range (IQR). A P value <0.05 was considered statistically significant. Based on the median, NLR values were divided into two groups. Differences in baseline characteristics in the diabetic and nondiabetic groups were compared using χ2 tests for categorical variables and an independent sample t test or ManneWhitney U test for continuous variables. An analysis of multivariate logistic regression was conducted to examine the association between the NLR and diabetes, and odds ratios (ORs) and 95% confidence intervals (CIs) were calculated. Correlation analysis was assessed by Spearman’s rank test.

## Results

There were 291 patients with pancreatic disease in our study and 32.3% (n=94) of them were diagnosed with diabetes mellitus. **Table 1** shows the biochemical and clinical characteristics of the patients. Age (P =0.002), FPG levels (P < 0.0001), HbA1C levels (P < 0.0001), white blood cell counts (P < 0.0001), NLR values (P=0.001), and MLR values (P=0.035) were higher for DM patients than for those without DM. Serum urea (P=0.007), albumin (P=0.004) and high-density lipoprotein (P=0.018) levels were significantly higher in patients with DM than in those without DM. In terms of BMI, PLR and NMR values, there were no significant differences between the groups.

**Table 1:** Patient characteristics according to diabetes mellitus (DM) status.

Variable	ALL (n=291)	Non-DM (n=197)	DM (n=94)	P
Disease category, n (%)				<0.0001
PBLT	100 (34.4)	83 (42.1)	17 (18.1)	
CP	45 (15.5)	27 (13.7)	18 (19.1)	
PDAC	146 (50.1)	87 (44.2)	59 (62.8)	
Age (year)	55.30 ± 13.23	53.61 ± 13.09	58.82 ± 12.89	0.002
Sex, n (%)				0.101
Male	165 (56.7)	105 (53.5)	60 (63.8)	
Female	126 (43.3)	92 (46.7)	34 (36.2)	
Body mass index (kg/m2)	22.13 ± 3.47	22.06 ± 3.40	22.26 ± 3.64	0.644
Fasting plasma glucose (mmol/L)	5.35 (4.71,6.77)	4.97 (4.61,5.54)	7.82 (6.17,9.90)	<0.0001
HbA1c (%)	5.90 (5.50,6.50)	5.70 (5.30,6.00)	6.90 (6.50,8.23)	<0.0001
Total bilirubin (umol/L)	10.90 (8.20,13.70)	10.80 (8.20,13.65)	11.10 (8.08,15.00)	0.584
Alanine aminotransferase (IU/L)	18.00 (12.00,28.00)	16.00 (12.00,25.50)	20.00 (12.75,33.25)	0.061
Albumin (g/L)	42.26 ± 6.79	43.06 ± 6.72	40.58 ± 6.66	0.004
Urea (mmol/L)	291.00 (245.00,351.00)	302.00 (254.50,355.50)	271.00 (223.00,339.25)	0.007
Creatinine (umol/L)	68.00 (57.00,78.00)	68.00 (57.00,78.50)	67.00 (56.00,75.75)	0.392
Triglycerides (mmol/L)	1.17 (0.87,1.59)	1.16 (0.87,1.54)	1.28 (0.80,1.80)	0.164
Cholesterol (mmol/L)	4.01 (3.50,4.63)	3.98 (3.51,4.54)	4.15 (3.48,4.77)	0.402
High density lipoprotein (mmol/L)	1.21 ± 0.36	1.24 ± 0.36	1.14 ± 0.35	0.018
Low density lipoprotein (mmol/L)	2.34 (1.90,2.87)	2.32 (1.90,2.80)	2.40 (1.82,2.94)	0.702
Total bile acid (umol/L)	3.80 (2.00,6.40)	3.80 (2.10,6.35)	3.60 (1.70,7.40)	0.770
Hemoglobin (g/L)	130.52 ± 15.63	130.00 ± 15.46	131.61 ± 16.02	0.415
White blood cells (10^9^/L)	5.52 (4.58,6.58)	5.35 (4.31,6.24)	5.87 (5.07,7.39)	<0.0001
Platelets (10^9^/L)	171.00 (134.00,214.00)	167.00 (126.50,217.00)	181.00 (144.75,214.00)	0.148
NLR	2.31 (1.68,3.01)	2.18 (1.65,2.77)	2.62 (1.80,3.59)	0.001
PLR	120.98 (93.94,146.00)	120.00 (92.27,142.85)	123.90 (95.56,148.97)	0.427
MLR	0.28 (0.21,0.37)	0.27 (0.21,0.34)	0.30 (0.22,0.39)	0.035
NMR	8.11 (6.61,10.30)	7.91 (6.36,9.77)	8.38 (6.94,10.82)	0.086

Significance level was set as P<0.05 (compared to the Non-DM group).

CP, Chronic pancreatitis; PDAC, pancreatic ductal adenocarcinoma; PBLT, pancreatic benign and low-grade tumor; NLR, neutrophil-to-lymphocyte ratio;

PLR, platelet-to-lymphocyte ratio; MLR, monocyte-to-lymphocyte ratio; NMR, neutrophil-to-monocyte ratio; HbA1c, Hemoglobin A1c.

**Table 2** shows the crude and adjusted correlations between the NLR and risk factors for DM. In the crude model, the unadjusted OR (95% CI) of DM was correlated with a gradual increase in the NLR value (OR: 1.383, 95% CI: 1.155-1.655, P<0.0001). In the final multivariate models, a gradual increase in the NLR value was an independent risk factor for diabetes secondary to exocrine pancreatic disorders (OR: 1.37, 95% CI: 1.138-1.649, P=0.001) after adjusting for covariates. On the other hand, higher values of albumin (OR: 0.94, 95CI%: 0.896-0.987, P=0.013), urea (OR: 0.996, 95CI%: 0.993-0.999, P=0.014 and high density lipoprotein (OR: 0.419, 95%CI: 0.187-0.941, P=0.035) were also as proved as predictors of DM among pancreatic disease.

**Table 2:** Risk factors for diabetes by univariate and multivariate logistic regression analyses.

Variables	Univariate analysis		Multivariate analysis	
	OR (95% CI)	P	OR (95% CI)	P
Disease category				
PBLT	Ref			
CP	3.255 (1.474-7.189)	0.004	3.593 (1.505-8.581)	0.004
PDAC	3.311 (1.785-6.141)	<0.0001	3.931 (1.991-7.765)	<0.0001
Sex				
Female	Ref			
Male	1.546 (0.933-2.563)	0.091	1.112 (0.599,2.066)	0.736
Age >55	2.176 (1.216-3.893)	0.009	1.813 (0.953-3.452)	0.070
Body mass index	1.017 (0.948-1.091)	0.643	NA	
Total bilirubin	1.023 (0.977-1.07)	0.334	NA	
Alanine aminotransferase	1.005 (0.996-1.015)	0.243	NA	
Albumin	0.941 (0.902-0.981)	0.004	0.94 (0.896-0.987)	0.013
Urea	0.996 (0.993-0.999)	0.005	0.996 (0.993-0.999)	0.014
Creatinine	0.992 (0.977-1.007)	0.303	NA	
Triglycerides	0.991 (0.935-1.051)	0.768	NA	
Cholesterol	1.023 (0.799-1.31)	0.855	NA	
High density lipoprotein	0.418 (0.201-0.868)	0.019	0.419 (0.187-0.941)	0.035
Low density lipoprotein	0.949 (0.796-1.131)	0.559	NA	
Total bile acid	1.000 (0.979-1.022)	0.986	NA	
Hemoglobin	1.007 (0.991-1.023)	0.413	NA	
Platelets	1.003 (0.999-1.007)	0.103	NA	
NLR	1.383 (1.155-1.655)	<0.0001	1.37 (1.138-1.649)	0.001
PLR	1.003 (0.999-1.007)	0.151	NA	
MLR	4.400 (1.115-17.364)	0.034	1.572 (0.42,55.198)	0.817
NMR	1.068 (0.993-1.149)	0.077	1.072 (0.903,1.272)	0.428

CP, Chronic pancreatitis; PDAC, pancreatic ductal adenocarcinoma; PBLT, pancreatic benign and low-grade tumors; NLR, neutrophil-to-lymphocyte ratio; PLR, platelet-to-lymphocyte ratio; MLR, monocyte-to-lymphocyte ratio; NMR, neutrophil-to-monocyte ratio; NA, not applicable.

**Figure 1:**
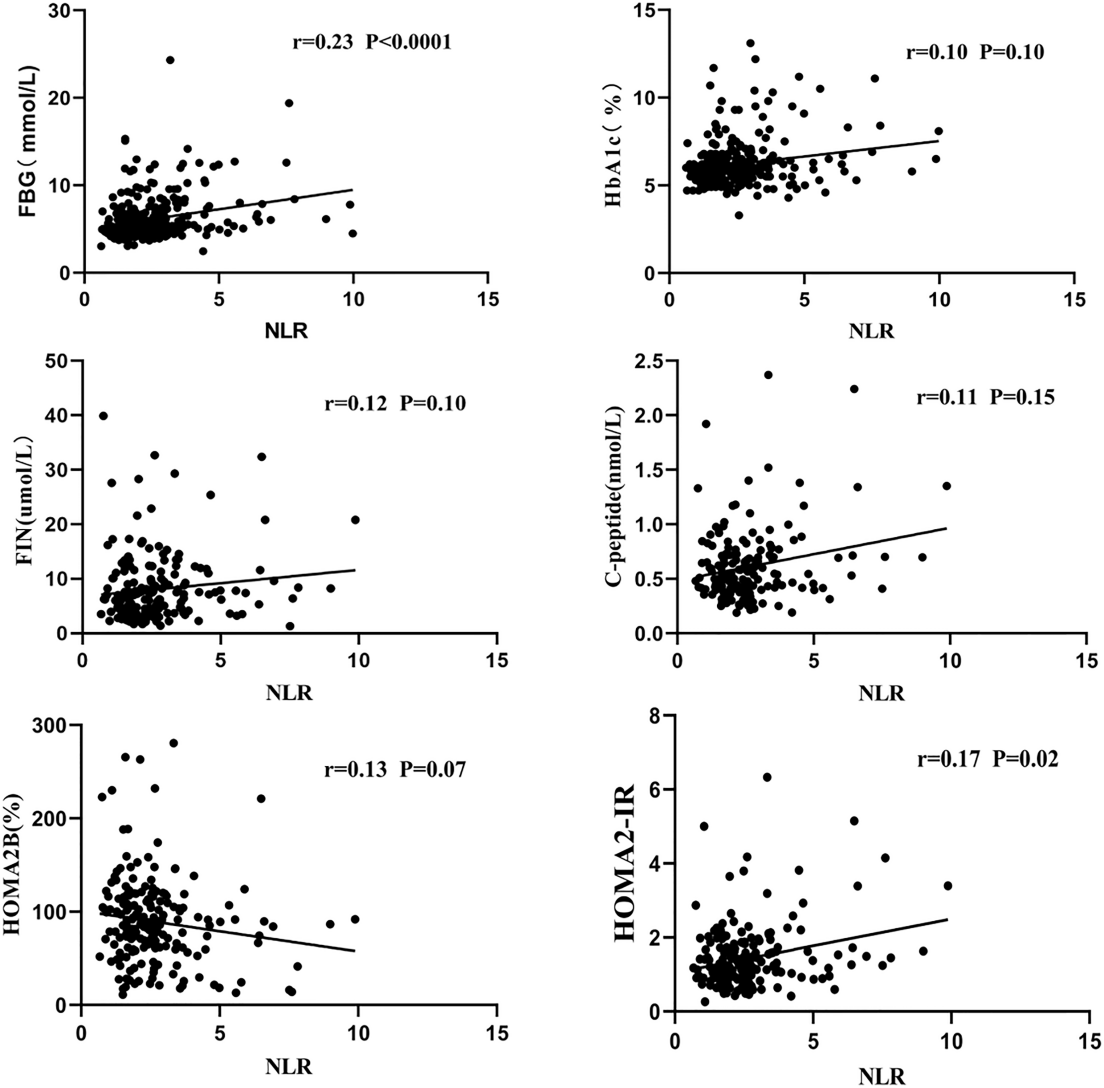
A scatter plot of the Spearman correlation analysis between the NLR and glucose-related index.

A scatter plot of the Spearman correlation analysis is shown in **Figure 1**. The NLR was significantly positively correlated with FBG (r = 0.23, P<0.0001) and HOMA2-IR (r=0.17, P=0.02).No significances were observed between NLR and HbA1c (r=0.10, P=0.10), fasting insulin (r=0.12, P=0.10), fasting C-peptide (r=0.11, P=0.15) and HOMA2-B (r=0.13, P=0.07).

The stratified analysis of the clinical and demographic variables based on the median NLR value (2.31) is shown in **Table 3**. More patients with PDAC, but fewer patients with PBLT, had higher NLR values. Patients with higher NLR values had higher fasting insulin (P=0.046), FPG (P=0.004) and HOMA2-IR values (P=0.028). Patients with a higher NLR value were more likely to have higher albumin and white blood cell levels. No significant differences in age, BMI, HbA1c, HOMA2-B, fasting c-peptide or liver and kidney function parameters were observed (P > 0.05 for all).

**Table 3:** Characteristic of the subjects stratified by values of the NLR.

Variable	Low NLR group (<2.31, n=145)	High NLR group (>2.31, n=146)	P
Disease category, n (%)			0.013
CP	55 (37.9)	45 (30.8)	
PDAC	29 (20)	16 (11)	
PBLT	61 (42.1)	85 (58.2)	
Age (year)	54.70 ± 13.62	55.88 ± 12.86	0.448
Sex, n (%)			0.098
Male	75 (51.7)	90 (61.6)	
Female	70 (48.3)	56 (38.4)	
Body mass index (kg/m2)	22.18 ± 3.48	22.08 ± 3.48	0.799
Fasting plasma glucose (mmol/L)	5.11 (4.65,6.13)	5.60 (4.87,7.59)	0.004
Fasting insulin (umol/L)*	6.13 (3.42,8.83)	7.30 (3.94,10.95)	0.046
Fasting c-peptide (nmol/L)^#^	0.50 (0.38,0.67)	0.57 (0.42,0.77)	0.131
HOMA2 β*	93.10 (65.95,119.23)	86.25 (61.08,112.48)	0.419
HOMA2 IR *	1.11 (0.89,1.66)	1.29 (0.94,1.89)	0.028
HbA1c (%)	5.9 (5.5,6.3)	6.0 (5.5,6.5)	0.424
Total bilirubin (umol/L)	10.60 (8.20,14.00)	11.40 (8.10,13.50)	0,969
Alanine aminotransferase (IU/L)	17.00 (12.00,28.50)	18.00 (13.00,28.25)	0.336
Albumin (g/L)	41.33 ± 6.77	43.17 ± 6.70	0.020^*^
Urea (mmol/L)	289.00 (249.00,338.50)	297.00 (241.00,359.00)	0.837
Creatinine (umol/L)	67.00 (56.00,76.00)	68.50 (58.00,80.50)	0.172
Triglycerides (mmol/L)	1.08 (0.84,1.58)	1.24 (0.89,1.61)	0.126
Cholesterol (mmol/L)	3.95 (3.44,4.54)	4.07 (3.55,4.72)	0.251
High density lipoprotein (mmol/L)	1.21 ± 0.35	1.21 ± 0.37	0.917
Low density lipoprotein (mmol/L)	2.28 (1.82,2.80)	2.45 (1.99,2.91)	0.126
Total bile acid (umol/L)	3.70 (1.90,6.45)	3.90 (2.05,6.20)	0.678
Hemoglobin (g/L)	129.18 ± 13.74	131.86 ± 17.25	0.144
White blood cells (10^9^/L)	5.12 (4.28,5.97)	5.98 (5.15,7.24)	<0.0001
Platelets (10^9^/L)	171.00 (132.00,213.00)	172.50 (134.75,219.75)	0.686

CP, Chronic pancreatitis; PDAC, pancreatic ductal adenocarcinoma; PBLT, pancreatic benign and low-grade tumors; NLR, neutrophil-to-lymphocyte ratio; PLR, platelet-to-lymphocyte ratio; MLR, monocyte-to-lymphocyte ratio; NMR, neutrophil-to-monocyte ratio.

*, 99 patients in the low NLR group and 98 patients in the high NLR group completed insulin- and β cell function-related index testing.

^#^84 patients in both the low NLR group and the high NLR group completed fasting c-peptide tests.

## Discussion

We present the first study demonstrating the link between the NLR and pancreatic diabetes and insulin resistance. In our study, we observed that patients with elevated NLR values that were associated with tumors or inflammation due to exocrine pancreatic diseases were more likely to develop diabetes. The prevalence of patients with diabetes secondary to exocrine pancreatic disease was higher in those with an NLR value > 2.31 than in those with an NLR value < 2.31. Moreover, HOMA-IR-measured insulin resistance was also positively associated with increases in the NLR. This finding indicates that a low-grade systemic inflammatory response may be considered an underlying factor that might contribute to the pathogenesis of pancreatic diabetes. While further research is needed to clarify this issue, we believe that elevated NLR values could be a significant predictor of diabetes secondary to exocrine pancreatic disorders.

Neutrophils respond to active, nonspecific inflammation as the first line of defense and can promote the chronic inflammatory state by recruiting macrophages and by interacting with antigen-presenting cells, whereas lymphocytes play a crucial role in the regulation and protection of inflammation (9). As opposed to the total WBC count, the NLR shows a dynamic aspect and has a better predictive value than the total leukocyte count (10). Although there are still many unknowns about the mechanisms behind the association between systemic inflammation and prevalent conditions, an increasing number of studies have established the utility of the NLR as a medically relevant biomarker. The NLR, as a repeatable and affordable biomarker calculated by peripheral blood, has been discussed as an innovative inflammatory biomarker to reflect the inflammatory status (11). Specifically, the NLR acts as an inflammatory factor, both by reducing the lymphocyte count and by increasing the neutrophil count.

However, the details of the mechanisms underlying the predictive value of the NLR are not entirely understood. Accumulated epidemiological evidence has identified that the inflammatory response is involved throughout the course of diabetes and cancer, and furthermore, systemic inflammation has been described as a potential pathogenic factor for the development of diabetes mellitus and its complications (3, 4). Recently, Wan et al. (12) also revealed that elevated NLR values are associated with a risk not only for cardiovascular and cerebrovascular diseases but also for diabetic kidney disease. In addition, a large number of studies have demonstrated the association between the NLR and pancreatic diseases, including PDAC and pancreatitis (7, 13, 14). A high NLR value plays an important role throughout the course of pancreatic cancer, and it could be a novel marker for survival evaluation and could help clinicians develop therapeutic strategies for pancreatic cancer patients (7, 15, 16). The NLR value alone or combined with the CA199 level could allow earlier diagnosis of PDAC in T2DM patients (16, 17). Dong et al. observed significantly higher NLR and PLR values in PDAC patients with T2DM than in patients with T2DM alone and healthy controls (17). Similarly, the NLR was used as a novel serum marker to predict the severity of acute pancreatitis and its adverse events (18, 19). Previous studies have revealed a significant association between CP and altered total leukocyte and lymphocyte counts (20, 21). Our study is in agreement with the previous results of NLR values among patients with exocrine pancreatic diseases regardless of their diabetic status, which indicate that elevated NLR values due to the existence of tumors or inflammation from exocrine pancreatic disorders were found to be associated with an increased risk for DM. The prevalence of diabetes secondary to exocrine pancreatic disease was higher in patients with an NLR value > 2.31 than in those with an NLR < 2.31. Together, growing evidence suggests that the NLR value is a significant predictor of diabetes secondary to exocrine pancreatic disorders.

T3cDM is heterogeneous and involves multifactorial underlying mechanisms consisting of the inflammation, fibrosis, and sclerosis of pancreatic tissue (2). The initial process of pancreatic disease, such as pancreatic cancer and pancreatitis, involves a multifactorial response involving the inflammation-mediated injury of endocrine and exocrine cells (18, 22–24). Previously, researchers showed a link between a high NLR value and varying degrees of glucose intolerance and insulin resistance in patients with T2DM (25–27). Shiny et al. observed that NLR values increased with the severity of T2DM and were positively related to HbA1c (r=0.411, P<0.0001), FPG (r=0.384, P<0.0001) and IR (r=0.233, P<0.0001) (26). Another study showed a significant positive correlation between the NLR and HOMA-IR (r = 0.285, P < 0.001). The IR odds ratio increased by a factor of 7.231 (95% CI, 4.277-12.223) for every one-unit increase in the NLR (26). The present study is in accordance with other reports that found that elevated NLR values were positively associated with high insulin resistance, which indicates that the NLR was significantly positively correlated with FPG levels and HOMA2-IR values. Although no significance was found between NLR and HOMA2-B, a trend that the negative correlation between the both was observed. The neutrophil effect may lead not only to insulin resistance but also to dysfunction of islet β cells and further studies should be performed to clarify it.

The details of the mechanisms underlying the association between the NLR and DM are not entirely understood, especially for T3cDM. Increased neutrophil levels may mediate IR in part through increased inflammation (28, 29). Increased NLR values appear to underlie the increased levels of proinflammation, as evident from the persistent neutrophil activation, hypersecretion of inflammatory factors, and enhanced release of neutrophil elastase, and induce insulin resistance, leading to subsequent overt diabetes (30). Moreover, the inflammatory environment might partly explain the β-cell dysfunction observed in chronic pancreatitis and PDAC. A previous study demonstrated increased inflammatory cell infiltration near islet β cells and reduced beta cell identity in chronic pancreatitis and PDAC (31, 32). In contrast, the effects of hyperglycemia on neutrophil apoptosis have been studied, which result in impairments in neutrophil clearance and prolonged inflammation in mice with diabetes (33). An increase in apoptosis has been documented both in rats with diabetes and in patients with diabetes, and elevated levels of oxidative DNA damage have been found in peripheral blood lymphocytes (34). Hyperglycemia due to DM is associated with the increased activation of leukocytes and their subtypes (35). In addition, hyperglycemia promotes the suppression of cytokine signaling action, thereby impairing insulin release and signaling cascades (36). This implies that improvement in glycemic control might suppress the inflammatory response, which could support the link between glucose metabolism disorders and inflammation. Future studies should focus on this pancreatic disease due to its important foundation for the development of pancreatic diabetes.

Limitations of the present study should be noted. One of the limitations of this study was that the determination of diabetes through HbA1c and fasting blood glucose levels might have affected the definition of diabetes and the analysis results. In addition, the present study had a retrospective cross-sectional design and did not allow us to investigate causal associations between glucose intolerance and the NLR. With a prospective design and multiple measurements of the NLR, future research should be able to provide strong evidence on the NLR as a subclinical inflammatory indicator for diabetes.

## Conclusions

In conclusion, the NLR is significantly increased in patients with diabetes secondary to exocrine pancreatic disorders and is associated with insulin resistance. NLR values may be reliable predictive markers for diabetes among patients with exocrine pancreatic disorders.

## Data Availability Statement

The original contributions presented in the study are included in the article/**Supplementary Material**. Further inquiries can be directed to the corresponding author.

## Ethics Statement

The study was reviewed and approved by the Medical Ethics Committee of West China Hospital at Sichuan University. The patients/participants provided their written informed consent to participate in this study. All study participants or their legal guardians provided informed written consent about personal and medical data collection prior to study enrollment.

## Author Contributions

GC and CT contributed equally to this paper. YC and XL designed the research. GC, CT, YC and XL performed research and analyzed data. GC, CT and YC wrote the manuscript. All authors read and approved the final manuscript.

## Funding

This study was supported by Research grants from the Key Research and Development Projects in Sichuan Province (2019YFS0043) and the 1·3·5 Project for Disciplines of Excellence, West China Hospital, Sichuan University (ZY2017302−1.3.5).

## Conflict of Interest

The authors declare that the research was conducted in the absence of any commercial or financial relationships that could be construed as a potential conflict of interest.

## Publisher’s Note

All claims expressed in this article are solely those of the authors and do not necessarily represent those of their affiliated organizations, or those of the publisher, the editors and the reviewers. Any product that may be evaluated in this article, or claim that may be made by its manufacturer, is not guaranteed or endorsed by the publisher.
